# Making sense of the delegitimation experiences of people suffering from indoor air problems in their homes

**DOI:** 10.1080/17482631.2022.2075533

**Published:** 2022-05-12

**Authors:** Tuija Seppälä, Eerika Finell, Suvi Kaikkonen

**Affiliations:** a Department of Social Sciences, University of Eastern Finland, Kuopio, Finland; b Department of Finnish, University of Helsinki, Helsinki, Finland

**Keywords:** Delegitimation, experience, illness, indoor air problems, making sense, thematic analysis

## Abstract

**Purpose:**

Little is known about the delegitimation experiences of people who associate their health problems with the indoor air quality of their homes (i.e., indoor air sufferers). From other contexts, it is known that people suffering from contested illnesses frequently report delegitimation from authorities and laypersons. Therefore, we analysed delegitimation experiences among indoor air sufferers, focusing on how they explain why others delegitimize them.

**Method:**

Two types of qualitative data—semi-structured interviews with eight people and essays written by 28 people—were subjected to a thematic analysis.

**Results:**

Thematic analysis revealed three themes: 1) *lack of understanding*; 2) *others’ lack of morality*; and 3) *social discrimination and inequality*.

**Conclusion:**

This study demonstrates that indoor air sufferers are vulnerable as individuals and as a group, and suggests that authorities working with people suffering from indoor air problems in homes must pay more attention to sufferers’ ability/willingness to trust people and the system responsible for their care.

## 1. Introduction

Problems in indoor air quality (e.g., the presence of indoor mould, chemicals) can affect people’s health in many ways, including causing asthma and other respiratory symptoms and infections (Jaakkola et al., 2013; Kanchongkittiphon et al., 2015; Mendell et al., 2011). There is also evidence that poor indoor air quality is associated with many non-specific symptoms, such as eye irritation, fatigue, and dermal symptoms (Smedje et al., 2017; Zhang et al., 2019). Despite their well-known adverse health effects, aetiology of indoor-air-related symptoms are partly contested, and psychosocial factors are also shown to contribute to these symptoms (Finell, Tolvanen et al., 2018; Magnavita, 2015). This makes indoor air sufferers (people associating their health problems to a specific building or buildings) especially vulnerable to belittlement, disbelief and other forms of delegitimation (Finell & Seppälä, 2018; Finell, Seppälä et al., 2018a; Söderholm et al., 2016). Delegitimation refers to the various ways in which sufferers experience their definitions or perceptions of their condition disconfirmed (Kleinman, 1992). Experienced delegitimation can negatively impact on health. For example, experienced delegitimation from institutions is found to strengthen the negative psychological health impacts of experienced chronic environmental contamination (see for a review Schmitt et al., 2021).

Delegitimation experiences are common among people suffering from contested illnesses, such as Gulf War syndrome (GWS), chronic fatigue syndrome (CFS), and multiple chemical sensitivity (MCS). Sufferers of contested illnesses often experience the existence of their illness being questioned or denied (Armentor, 2017; Dickson et al., 2007; Finell & Seppälä, 2018; Nettleton, 2006). Their symptoms are perveived to be psychosomatic, and they are offered psychological diagnoses (Finell & Seppälä, 2018; Glenton, 2003; Kornelsen et al., 2016; Lillrank, 2003; Ware, 1992); their motives are questioned and their needs are unrecognized (Dickson et al., 2008; Montali et al., 2011; Richardson, 2005); and their rights to compensation and care are denied (Dumit, 2006; Finell & Seppälä, 2018). Experiences of this kind can illustrate epistemic injustice: epistemic asymmetry between the people suffering from contested illness and the decision-making authorities (e.g., physicians) can lead the authorities to unrecognize and discredit suffers’ knowledge for instance, because of their distrust in sufferers (Blease et al., 2017; Buchman et al., 2017; Fricker, 2007).

Although extant research shows that delegitimation is manifested on various levels, less is known about how people suffering from contested illnesses explain why they are delegitimized by other people. Some research shows that sufferers attribute perceived delegitimation to others’ lack of information or knowledge (Armentor, 2017; Kornelsen et al., 2016), the invisibility of symptoms (Dickson et al., 2007; Montali et al., 2011), and the lack of official recognition of their disease (Dickson et al., 2008). Research on this topic is needed. For example, there has been no systematic analysis of the different ways that sufferers explain the behaviour of those they perceive to be delegitimizing them and what these explanations reveal about their experiences of being delegitimized. Such an analysis is needed because studies conducted in various contexts have shown that how peoples explain their negative experiences has long-lasting consequences for their well-being (Chae et al., 2011; Crocker & Major, 1989; Martinko et al., 2012; Park & Baumeister, 2017). For example, interpretations of the physical and social stressors caused by an illness influence how people adapt to that illness (St Claire, 2003).

### 1.1. The present paper

Little is known about the delegitimation experiences of people who associate their health problems with the indoor air quality of their homes. In this qualitative study, we explore delegitimation experiences reported by such sufferers, focusing especially on their explanations of why others delegitimize them. It is especially important to analyse this issue in this context because a home should be a space where people feel they are safe and sheltered: when this is not the case, the experience can be very stressful, and people can feel threatened (Shenassa et al., 2007; Suglia et al., 2011). This knowledge is also very important for various stakeholders, particularly healthcare professionals and other authorities, whose responsibilities include helping such sufferers and building trust with them.

Our sample population was recruited from residents of Finland, where approximately 6–11% of the population are exposed to daily moisture and mould damage in their homes (Reijula et al., 2012). The official healthcare system does not recognize a causative link between poor indoor air quality and health problems because the factors and mechanisms explaining such health effects are unknown (Current Care Guidelines, 2017). This makes Finland a relevant context for studying people’s delegitimation experiences related to indoor air problems at home.

## 2. Methods

### 2.1. Sampling and respondents

This study is based on two data sets. The first one is a subset of data from a large *interview-study* and one from a large *essay-study*. To recruit respondents for the interviews, an announcement was released on a website and a Facebook group of the Organization for Respiratory Health in Finland, and in a magazine published by the same organization. Over 100 individuals responded, expressing a willingness to be interviewed about their indoor air-related experiences. From this pool, 30 individuals were selected and interviewed to represent a cross-section of contexts (i.e., a home, a workplace, an educational establishment), different regions of the country, and different phases of illness (i.e., able to work, on sick leave, permanently unable to work) and to include both men and women (6 men and 24 women were selected). From these interviews, we selected those in which respondents identified indoor air problems in their homes as the cause of their symptoms or illnesses (four men and four women, aged between 26 and 63 years), and left out the interview, which described indoor-air-related experiences in other contexts (i.e., a workplace, an education establishment). Three of the respondents had a diagnosis of asthma, one had an MCS diagnosis and three had diagnoses for allergies. Two of the respondents had indoor air problems in a house they currently or previously owned, one had indoor air problems in an apartment she owned, and five respondents had/have had problems in a house they rented.

To recruit respondents for the *writing event* (the *essay-study*), announcements were released via non-profit public health organizations, in magazines, and placed in online news sites by the Finnish Literature Society (SKS), which organized the event in collaboration with the second author of this paper in 2014. A total of 62 essays were received in which participants described their experiences with indoor air problems. We selected essays by 28 individuals who identified indoor air problems in their homes as the cause of their symptoms or illness and left out the essays which described indoor-air-related experience in other contexts (i.e., a workplace, an educational establishment). These essays were written by two men, 21 women, and five individuals who withheld their gender; they ranged in age from 25 to 85 years old.^1^ Of the 17 participants who provided information on home ownership, eight were owner-occupiers, two lived in a house owned by their spouses, and seven lived in a rented home. The participants associated a variety of health problems with poor indoor air quality, ranging from common medical conditions such as asthma, sinusitis, and bronchitis to various symptoms affecting multiple organs, such as respiratory symptoms, headaches, dermal, intestinal, and neurological inflammation, and other problems. The reported problems ranged from mild and short-term to severe and chronic.

### 2.2. Procedures

#### 2.2.1. Interviews

All respondents provided informed consent for their transcripts to be used for research purposes. Interview protocol and interview questions were planned by two researchers working at the Organization for Respiratory Health in Finland who also conducted the face-to-face interviews based on a semi-structured format in 2013. At the beginning of each thematic interview the respondents were encouraged to tell their stories freely; more focused and sensitive questioning was introduced as the story took shape. The respondents were asked about their experiences of indoor air-related symptoms, their homes, their social relationships, and their interactions with authorities. Interviews did not include direct questions about respondents’ delegitimation experiences. The interviews were transcribed verbatim. The length of the transcriptions used in this study varied from 4,651 to 11,586 words. The data were anonymized before being delivered to the authors of this study.

#### 2.2.2. Essays

In the announcement, the respondents were informed that their essays would be used as research material and archived by the SKS, which is a research institution and a national memory and cultural organization. The respondents were asked to write about their experiences relating to indoor air problems and were directed to a series of stimulus questions available on the SKS website (see questions in Finell & Seppälä, 2018, supplementary material). The respondents were instructed to post their stories or save them on the SKS website. The stories varied in length between 104 and 7,546 words. An official from the Tampere Region Ethics Committee approved both stages of this study.

### 2.3. Analysis

We conducted a hybrid of deductive and inductive thematic analysis (Braun & Clarke, 2006; Fereday & Muir-Cochrane, 2006). The analysis started with the interview data; we then analysed whether the same and/or additional themes could be identified in the essay data.

After carefully familiarizing ourselves with the interview data, we identified all the instances where respondents described how and why someone had mistreated them or someone else who suffered from indoor air-related problems at home. Any accounts that were not associated with a respondent’s explicit or implicit explanations of another individual’s behaviour were excluded from the final data set, as we were specifically interested in these explanations (Braun & Clarke, 2006). In very rare cases, the respondents partly blamed themselves for others’ behaviour (e.g., being too kind, trusting or gullible); because there were only a few short statements, these were left out of the final data set.

We analysed the final data set inductively by following the five phases of thematic analysis described by Braun and Clarke (2006). In this analysis, we focused on how the respondents made sense of their delegitimation experiences. Three themes were developed in the analysis of the interview data (Braun & Clarke, 2006). In the essay data set, we directly identified all the instances in which respondents explained why someone had mistreated them or someone else. We compared these explanations with those identified in the analysis of the interview data. The three themes developed in the analysis of the interview data were also relevant for the instances identified in the essay data, and we did not develop any new themes.

## 3. Results

We developed three themes depicting the respondents’ explanations for why others delegitimized their experiences and perceptions. The first theme was the respondents’ perception that others lacked knowledge and understanding, the second was the respondents’ disappointment with others’ morality, and the third was the respondents’ concerns about discrimination and social inequality. We explain these three themes in detail below.

### 3.1. Theme 1: lack of understanding

The first theme represents the respondents’ perception that they were delegitimized by others due to a lack of knowledge and understanding. The respondents thought that such a lack of understanding was related to the invisibility of their symptoms, uncertainty regarding the aetiology of symptoms, and laypersons’ and authorities’ lack of experience and knowledge. Perceptions of these kinds have been identified also among people suffering from other contested illnesses (Armentor, 2017; Dickson et al., 2007, 2008; Kornelsen et al., 2016; Montali et al., 2011). Laypersons’ perceived lack of understanding was an ambivalent experience for the respondents: on the one hand, they understood others’ struggles, but on the other hand they hoped that others would have just believed them, which could have helped them to maintain their moral character (see Åsbring & Närvänen, 2002). Authorities’ lack of understanding was an empowering experience for some and a threatening experience for others.

Even though respondents often perceived the reason for delegitimation to be the contested nature of indoor air problem itself, they still expected their physicians to understand how invalidating the symptoms could be. This understanding was not always received, as one respondent wrote, “general practitioners belittle these symptoms” (Essay 26). Another respondent felt that doctors should be humbler regarding the limits of their knowledge, explaining:

I am an experience-based expert; I am not a [medical] professional [in this field] …. Nevertheless, I really believe that somehow [physicians] still work unprofessionally. If we think in terms of the whole […], we live a training phase. […] As a professional, you should be quite humble when you are [giving] unconditional opinions, like “it can’t be this” or “it can’t be that” or “it does not look like …” and so on. (Interview 1)

In addition to demonstrating how the respondent demanded humility from physicians (“you should be quite humble”), this excerpt also illustrates a kind of power shift between experience-based experts and professionals in that the professionals were perceived as lacking understanding. This respondent defined himself as “an experience-based expert,” emphasizing that he was not a “professional.” However, he simultaneously questioned the authority of professionals by stating that “they still work unprofessionally.” This kind of power shift between professionals and laypeople is identified in the earlier research (Dickson et al., 2007) and was also evident in the way other respondents described their reactions: because of their physicians’ perceived incompetence, the respondents had extended their own knowledge and had withdrawn from medical care services, resorting to alternative medicine or trying to take care of themselves. One respondent described treating his eczema on his own:

I was prescribed hydrocortisone creams for years, and [eventually] I got a kind of double skin. […] I was reading a lecture handout from an English university about skin diseases, and then I thought, wow! […] I realized I could do something by myself … I peeled off that double skin and the crater in my skin with a scalpel. (Interview 2)

The respondent described realizing that he was not dependent on professionals and that he could treat himself in a professional way by utilizing their knowledge (“a lecture handout”) and tools (“a scalpel”). Another respondent wrote about her strategy against delegitimation: “I started active, surveying and solving housing health problems. Since I had also established my knowledge by studying, there has been no attempt to belittle me.” (Essay 8). However, the perceived incompetence of professionals was not an empowering experience for all respondents: “I consulted a couple of gynaecologists. As you could expect, for at least one of them suspected I had hormonal ailments. I did not believe her. I consulted a homeopath; I lost my money again. No explanation [to my symptoms]” (Essay 3). Some respondents reported being dependent on doctors who, they felt, did not understand their concerns, which threatened their sense of control. As one respondent put it:

It is just the worst when you are the best “doctor” and “indicator” [of your illness]. […] If you go somewhere there [and explain] “I need a doctor … I can’t control this thing anymore” […] others just dismiss you, [saying] that this “spring flu” is floating around, take some painkillers. (Interview 3)

The respondents expressed more empathy for laypersons’ lack of understanding: “Living with a sick and tired person was not always fun” (Essay 48). The respondents were aware that uncertainty also caused frustration in people who tried to help them, turning their initial support and empathy into disbelief and hostility (see also Dickson et al., 2007). One respondent described his experiences of his brother’s frustration and fluctuating support:

Sure, he has sometimes helped with moving and so on, but of course … when this goes on and on, those people lose their faith that helping is actually helpful. Perhaps we bickered a bit at some point, but that is … past now. Then, surprise, surprise … [brother’s] youngest son started to [have the same] symptoms […]. It has probably made the [illness] more real for them as well. Of course, you hope that it will not become real in that way. (Interview 4)

The respondent showed understanding for his brothers’ disbelief, but at the same time he seemed to feel some satisfaction when his brother’s lived experience advanced his understanding of his (the respondent’s) situation. Several respondents mentioned that true understanding can only be achieved by lived experience (see also e.g., Armentor, 2017). When relatives and friends did not have such an understanding, it could be received from other indoor-air-sufferers: “[My friends] changed to understanding others who [also] had indoor air problems” (Essay 18). However, the excerpt from Interview 4 above illustrate how this view is associated with ambivalent feelings; it is morally wrong to wish negative experiences for other people, but without these negative experiences, it would not be possible to understand them.

### 3.2. Theme 2: others’ lack of morality

The second theme represents the respondents’ experiences of authorities’ low morality. Respondents seemed to think that the authorities actually knew that respondents’ perceptions and definitions of indoor air problems were true, but because they conflicted with the authorities’ own interests (e.g., money and reputation), the respondents were delegitimized. One respondent said that “no doctor would testify that the disease is caused by mold, although they quite well knew that [was]” (Interview 2). Such responses indicate that the respondents felt that they were victims not just of indoor air problems, but also of others’ immorality; their intentional engagement in immoral behaviour such a lying and neglect of their duties such as care. These experiences were associated with feelings of unfairness. Those others who engaged in such delegitimation were perceived as opponents and respondents felt no compassion for them, as they did in Theme 1. Unlike in Theme 1, the others responsible for the delegitimation were always in positions of authority.

Many respondents accused authorities of selfishness, heartlessness, and lack of interest: “When a matter or problem doesn’t touch that person or their loved ones/relatives, (s)he is not ready to lift a finger to influence and help” (Essay 37). In the excerpt below, a respondent explains her experiences with a housing cooperative whose chairman denied that her symptoms had anything to do with her apartment and downplayed any suggestion that there were problems with the indoor air quality:

I realized that people who are totally cold from their heart, case-hardened … are unable to do the right things just because they are right. Instead, they only look at money, and they think that someone is just trying to use them …. It is impossible to expect the right behavior of them. It is like asking a crippled person to run a marathon. … I was very relieved when I realized that I can’t demand anything from a person who can’t give it. Irrespective of that, I needed to forgive all the anger and bitterness so that I could move on. (Interview 3)

Here, the respondent explicitly stated that authorities do not do the right things (in her case, repairing the apartment) but instead think only about the costs. In the context of care, experiences of this kind have been explained by a double-role of a physicians as a care provider and a gatekeeper of costs (Dumit, 2006). However, here (Interview 3) the respondent explained that these delegitimizing actions reflect the authorities’ inability to behave morally, which she emphasized by using the metaphor of “a cripple.” She attributed this disability to delegitimizers’ emotional characteristics by describing them as “cold from their heart” and “case-hardened.” There is clearly a tone of moral anger in the voice, and the respondent highlighted her moral superiority over delegitimizers by telling of her need to forgive these perpetrators. This moral anger may reflect not only the respondent’s feelings of unfairness but also her concern for her own morality in the eyes of others, as she was convinced that others tended to think that someone demanding repairs was “just trying to use them.” Like not having a medical diagnosis limits legitimate access to the sick role threatening patient’s sense of being a moral individual (e.g., Frank, 2013; Nettleton, 2006), in this case (Interview 3), not having legitimate evidence of the causality between apartment’s indoor air quality and respondent’s health problems limits the legitimacy of her demands threatening respondent’s sense of being a moral individual.

Several respondents experienced that the authorities prioritized wrong issues: “People’s health should be the priority instead of money and greediness and profit-seeking” (Essay 37). Besides money, several respondents accused authorities of caring more about their own reputation than they did about the respondents’ well-being. This accusation reflected the contested nature of indoor air problems:

Several physicians have said that they give good advice and that I should move out of there. However, in my medical statements, nothing like that ever appears … I have heard … that sometimes when physicians have given advice or tried to help, after a while they must tell their patients that they have to withdraw because otherwise they would lose their post. What kind of morals does that demonstrate? (Interview 4)

In this excerpt, the respondent describes how physicians support patients in private but then withdraw to protect themselves in public. These physicians’ morality is explicitly questioned at the end of the excerpt. It follows that the respondent felt that he was eventually left alone with his problems; as he explained later in the interview, “they could not nor did want to help me” (Interview 4). Another respondent wrote: “People are afraid of becoming labeled hysterics. The same is still observed in the activities of the authorities. They are more critical on these issues than they are with any other group of issues” (Essay 8). Prior research has demonstrated how physicians publicly defending patients suffering from contested illness can be stigmatized by their professional community (Phillips, 2010).

Even more intentionality was attributed to the authorities’ behaviour when they were accused of lying. In the excerpt below a respondent describes how his physician intentionally hid valuable information relating to his blood tests and belittled his assertions about the severe symptoms he was experiencing. According to the respondent, these tests revealed that he had, in fact, been exposed to various moulds.

An example of … how rude the lying is … so here are my blood tests and what was found [the subject takes a paper out]. The leading municipal physician said that there was only a little *Aspergillus pusillus* and whatever this is. However, he did not say that there was also evidence of exposure to *Aspergillus fumigatus* … Fusarium, Ulocladium, and … *Stachybotrys chartarum*. The bloody worst molds … he claimed there is nothing. And when I asked for these papers, I had to give a real primitive reaction, “Now give them to me, you Satan!”, the way they do in the bigger cities. This trying to hide [the truth]—the worst would not have been revealed, penicillium as well. (Interview 2)

The affective language used by this respondent suggested that he was still upset by the experience during the interview: “I started to feel an infernal hate towards that systematic lying” (Interview 2). Later in the interview, he claimed that his physician had caused his illness to become chronic and expressed his intention to seek revenge. His expressions of anger, bitterness, and hate were exceptionally strong, but other respondents also reported or demonstrated having these emotions: “I am angry […] is it legal that they can just rent out this kind of [an apartment] and no one cares” (Interview 5). They were responses to how unfairly they felt that they had been treated by the authorities. These strong negative emotions also gave the respondents the energy to defend themselves and their rights as reported by one respondent: “I was so angry … [I said to the board of the housing company] that if they do nothing to this [poor air conditioning] now, I will contact [the communal] health authority. So, the [adjustment of the air conditioning] was [conducted] the next week” (Interview 3).

### 3.3. Theme 3: social discrimination and inequality

The third theme captures the respondents’ experience that they and other indoor air sufferers are delegitimized because Finnish society discriminates against them. For example, they reported that the social security system seemed to discriminate against them more than it did other patient groups. Some respondents also reported that indoor air problems often affected disadvantaged groups (e.g., those with low socioeconomic status), and therefore society is indifferent towards them. Finally, some respondents felt that society treats different groups of indoor air sufferers unequally (e.g., different socioeconomic status and gender); several respondents seemed to believe that when well-off citizens (e.g., high socioeconomic status) had indoor air problems, they faced less delegitimation than disadvantaged citizens. Respondents experienced that the authorities were less willing to trust the knowledge of the indoor air sufferers who belonged to disadvantaged groups such as women, single parents and those living in a rented apartment illustrating epistemic injustice within the group of people suffering from indoor air problems (Fricker, 2007). Notably, even respondents who belonged to high-status groups perceived that delegitimation was related to the structural inequality in Finland’s society and wealth. For example, homeowners were thought to face less delegitimation than non-owners. One respondent wrote, “the municipal health inspector treated us well when the indoor air problem concerned our own townhouse, but when it later concerned a rental apartment owned by the municipality, the health detriment statement was not easily given” (Essay 26). These statements reflected both social distrust and solidarity with other indoor air sufferers. A respondent who received a disability pension and lived in a right-of-occupancy-apartment described his thoughts on discrimination and inequality as follows:

No one seriously wants to believe in this kind of illness … I think that these officials ought to be caught flat-footed, to show what kind of … outrageous game they are playing with us … and then they get a good salary. If they happen to move into “a sick building”, they can just move to another one with that good salary. However, for those who live in rented apartments or in right-of-occupancy apartments or those who pinch and save in an owner-occupied apartment purchased on credit, their situation is totally different. (Interview 4)

It is notable in this excerpt that the respondent refers to both indoor suffers and delegitimizers in the plural—“us” and “officials”—which suggests that delegitimation in environmental issues is associated with broader societal divisions and hierarchies (see e.g., Pellow, 2000). The indoor air sufferers interviewed associated their experiences of structural discrimination with distrust of authorities and institutions, which they felt were not on their side:” It was easy for officials and authorities to hide behind the organizations they represent” (Essay 8). Respondents’ distrust varied from mild doubt (“I guessed that there was something [wrong] because they didn’t hand [the inspection report] over to me”, Interview 5) to conspiracy theories and strong feelings that nobody could be trusted (“ … the other thing was … this nationwide plot among physicians to keep people sick in order not to reveal that [some people] have mold-related illnesses”, Interview 2).

In addition to socioeconomic status (e.g., home ownership, professional status), the respondents perceived indoor air sufferers’ unfair treatment to be related to gender (see also e.g., Armentor, 2017), as represented in the following excerpt:

I tried to explain that the water had leaked from under the piping to the inspection pit in the alcove. That information did not get into the heads of the menfolk, anyway. There is no need to care about the talk of the womenfolk. (Essay 60).

Different social categories (e.g., female gender and low socioeconomic status) also intersected in the descriptions, as in the story excerpted below. The respondent described how an acquaintance of his, a single mother living on social benefits, was not trusted and did not receive support from social services when she moved out of a municipal apartment that had indoor air problems:

The municipality refused [to give her a new apartment] because [administrators] claimed … that she moved out just because she wanted … a nicer house, [and administrators insisted] that there was nothing wrong with the former apartment. [This happened] irrespective of laboratory samples and evidence of about ten types of mold … dampness-related mold. (Interview 2)

It is notable in the data that even the respondents who were well-off themselves described their perceptions of such status-based differences in indoor air suffering. One of the respondents mentioned that the house she bought with her husband developed what appeared to be dampness and mould, so they thought they had to move out. They tried to clean the mould from their furniture with a chemical that turned out to be toxic. She found that some other indoor air sufferers were also aware that this chemical was causing problems. One of them, a single mother living in a small city, had tried to warn the authorities about the dangers of this chemical for three years, but they did not react until the respondent’s husband—a professor and a physician—contacted them, as she described in her interview:

My husband, he is a professor, a physician, called … and then it didn’t take many weeks until warnings [were placed on the chemical]. I have also cried because [the authorities] do not listen to a single parent from [a small city]. (Interview 6)

This excerpt illustrates the respondent’s experience of privilege, as well as her sense of solidarity with the lower-status woman. A similar experience of privilege was reported by another well-off respondent, who explained that he had moved out of the house he owned and had lived in with his son because it had mould, which affected him but not his son. In the following excerpt, he describes how he managed to convince an authority during a phone call that his son should not participate in mandatory military service because indoor air problems are common in army barracks and were a risk to his son’s health. He was an older male physician, and he seemed to attribute to his high status the understanding he received from an authority towards his struggles. He recognized that not everyone who suffers from indoor air problems had equal opportunities to improve their situation, as illustrated in the rhetorical question he asked while describing his phone call to the authority in the military service: “Okay, who has these kinds of opportunities? I called [name of a place], and I [spoke to] a very understanding [authority]. He said, that by no means [my son needs to participate in military service]” (Interview 1). The rhetorical question “who has these kinds of opportunities?” can be interpreted as a claim that rare people have opportunities of the same kind as he had to influence decision-makers.

## 4. Discussion

This study analysed how people suffering from indoor air problems in their homes explain their delegitimation experiences. To our knowledge, this is the first study that focuses on explanations of the delegitimation of individuals who associate their symptoms and illnesses with buildings and the first study that focuses systematically on delegitimation explanations in the context of contested illnesses. We identified three themes that described the various ways in which the respondents made sense of their distressing experiences with family members, friends, and authorities: lack of understanding, others’ lack of morality, and social discrimination and inequality.

These three themes reveal the vulnerability our respondents experienced at both the individual and group levels. At the individual level, when the respondents perceived that others did not understand the origin of their symptoms, the respondents demonstrated understanding and compassion towards these others’ lack of understanding (Nettleton, 2006). However, the respondents still needed help and care. When they felt their physicians were unable to provide proper care, the respondents reported experiences of losing control (Whelan, 2007) or tried to cope by resorting to self-care and consulting with alternative health practitioners who offered compensatory healing experiences (e.g., agency, empowerment, and recognition; Dickson et al., 2007; Sointu, 2006; Swoboda, 2006). Withdrawal from professional care illustrates respondents’ distrust in professionals, which might has developed from the professionals’ initial distrust and delegitimation of respondents’ understanding and perceptions of their conditions (Blease et al., 2017; Buchman et al., 2017).

Descriptions of authorities’ behaviour and lack of morality were not associated with any goodwill towards the perpetrators. Although the respondents perceived that the contested nature of their illness affected the authorities’ behaviour, they still felt that the authorities had a moral obligation to take care of them. When the expected care was not received and they felt that the authorities were not on their side, the victimhood associated with suffering from poor indoor air quality was enlarged to include authorities’ indifference, selfishness, and self-interest (see Dumit, 2006, on the double role of physicians). These feelings of unfairness were associated with descriptions of attempts at forgiveness, as well as desire for revenge and the deepening of distrust in authorities’ willingness to help. Previous literature suggests that suffering from a contested illness threatens patients’ sense of being moral individuals (Dickson et al., 2007; Frank, 2013; McParland et al., 2011; Nettleton, 2006). Thus, by ascribing the delegitimation they faced to a lack of morality on the part of the authorities involved, the respondents reversed the typical frame in what can be interpreted as attempts to preserve their own morality and agency.

The indoor air sufferers surveyed in this study also experienced vulnerability at the group level; they perceived that delegitimation was related to social discrimination and that it was particularly directed at socioeconomically disadvantaged groups, reflecting inequalities in social structures (e.g., divisions between poor and rich, healthy and sick, owners and tenants, and men and women). This explanation was associated, on the one hand, with expressions of deep social distrust in a system that was designed and expected to take care of their problems, but on the other hand with a sense of solidarity with other indoor air sufferers. Distrust and conspiracy theories can be particularly harmful to people coping with illness and chronic symptoms, especially those who are socially excluded or marginalized (Kramer, 1998). However, group-level attributions can also be psychologically rewarding, as a shared sense of identity may work as a buffer against stressors (Haslam & Reicher, 2006). Identification with others having similar experiences can also support collective action against experienced injustices (e.g., Dumit, 2006; Shriver et al., 2002).

To our knowledge, prior research on people suffering from indoor air problems has not recognized these explanations. In other contexts, experienced delegitimation is attached, for instance, to sexism (e.g., Armentor, 2017). It is possible that experiences related to social inequality and discrimination are related to the specific context of this study. Social inequality could be salient when people talk about their homes because one’s living arrangements are associated with one’s socioeconomic status (Evans & Kantrowitz, 2002). Furthermore, the quality of low-income housing compared to standard and luxury housing is reportedly worse in Finland and many other countries (Baker et al., 2016; Kauppinen et al., 2015), which may increase the salience of social divisions in this context. These findings highlight the importance of paying attention to possibly diverse experiences of different groups (e.g., gender, socio-economic status, race) among people suffering from a specific contested illness.

The results make clear the great extent to which indoor air sufferers perceive their fundamental needs of health, safety, and care are being delegitimized. Consequently, they deem their position vulnerable and the result of injustice (see also Finell & Seppälä, 2018), and they expressed deep distrust of the authorities they felt were failing to meet their duty of care. Being vulnerable may increase individuals’ risk of perceiving other people’s actions as manifestations of ignorance or maliciousness, which may amplify a vicious cycle of distrust and bitterness (Kramer, 1998). It also imposes a significant barrier to sufferers coping with their condition (St Claire, 2003). It is important that authorities bear in mind the elevated risk of miscommunication in the context of contested illnesses. What may be considered routine conduct from the perspective of an authority’s institutional agenda could be perceived as invalidating or indifference by an individual whose symptoms are not officially acknowledged. Therefore, we want to suggest that authorities not only validate these sufferers’ difficult situations and treat them empathetically but also make explicit the affordances and constraints of the assistance they can provide given their institutional position. In other words, authorities could be open about what forms of assistance they are able to provide and to what extent. More importantly, they could also make the institutional grounds of their decisions available to these sufferers, which could reduce the likelihood of being perceived as malicious and unwilling to provide care in light of the considerable discrepancy between a sufferer’s need for help and an authority’s limited resources, options, and knowledge.

## 5. Conclusion

Indoor air problems at home can seriously damage people’s sense of security. The contested nature of indoor air problems and their possible health effects can lead to sufferers feeling that authorities are not on their side and that they are left to survive alone. Distrust in authorities’ ability or willingness to help can also decrease sufferers’ willingness to cooperate and can marginalize them. Thus, special attention should be given to communication with these people, and practical solutions should be implemented to enhance their trust in the people and the system responsible for their care.

## Data Availability

The data are not publicly available.

## Note

1. Thirteen participants reported their age.

## Permission to reproduce material from other sources

No material from other sources used.
